# Role of Lower Esophageal Squamous Cell Carcinoma Margin Location on Abdominal Lymph Node Metastasis Risk

**DOI:** 10.3390/jcm12072657

**Published:** 2023-04-03

**Authors:** Xia Zhong, Xue-Hua Tu, Gu-Ha A-Lai, Ze-Guo Zhuo, Peng Yao, Ying Zhang, Zhi-Jie Xu, Yi-Dan Lin

**Affiliations:** 1 Department of Thoracic Surgery, West China Hospital, Sichuan University, Chengdu 610041, China; 2 Anesthesia Operation Center, West China Hospital, Sichuan University, Chengdu 610041, China; 3 West China School of Nursing, Sichuan University, Chengdu 610041, China; 4 Department of Pathology, West China Hospital, Sichuan University, Chengdu 610041, China

**Keywords:** esophageal cancer, assessment effectiveness, prognosis, lymph node metastasis, LED

## Abstract

Background: Different sites of esophageal cancer are accompanied by different regional lymph node metastasis (LNM) risks. We aimed to investigate the impact of a lower tumor margin on abdominal LNM risk. Methods: We enrolled patients who underwent esophagectomy for esophageal squamous carcinoma (ESCC) from 2014 to 2017 in West China Hospital. Overall survival (OS) analysis was performed. We measured the distance between the lower tumor margin and esophagogastric junction (LED) with upper gastrointestinal contrast-enhanced X-ray (UGCXR). Multivariate logistic regression analysis and propensity score matching (PSM) were performed to explore the relationship between LED and the risk of abdominal LNM. Abdominal LNM risk in ESCC was stratified based on the location of the lower tumor margin. A model predicting abdominal LNM risk was constructed and presented with a nomogram. Results: The included patients had an abdominal LNM rate of 48.29%. In multivariate logistic regression analysis, LED was identified as a risk factor for abdominal LNM. Subgroup analysis of middle ESCC showed that patients with an LED less than 10 cm had a significantly higher rate of abdominal LNM than those with an LED greater than 10 cm. The abdominal LNM rate in middle ESCC patients with an LED less than 10 cm was 32.2%, while it was 35.1% in lower ESCC patients whose lower tumor margin did not invade the esophagogastric junction (EGJ), which was comparable after PSM. Conclusions: LED could help surgeons evaluate the risk of abdominal LNM preoperatively and better guide dissection of abdominal lymph nodes according to risk level.

## 1. Introduction

Esophageal cancer is the eighth leading cause of cancer-related death worldwide [1]. Surgery is the most important treatment for locally advanced disease. In addition to resection of the tumor itself, lymphadenectomy is an essential step to achieve a cure [2,3]. Although the standard for the extent of lymphadenectomy differs worldwide, tumor location is the most important factor that surgeons take into consideration when making the decision [4,5]. The upper margin of the tumor is used to define tumor location in the 7th edition of the American Joint Committee on Cancer staging criteria for esophageal cancer [6]. However, it changed to the tumor center in the 8th edition [7]. However, neither of the staging criteria takes the lower margin into consideration. The lymph node drainage of the esophagus is bidirectional [8]. The upper and lower tumor margins indicate upward and downward invasion of the tumor, respectively. However, the lower tumor margin seems to be overlooked. Now that the upper tumor margin plays an important role in whether supraclavicular or cervical lymph node dissection should be performed, could the lower tumor margin be a predictor of abdominal lymph node metastasis?

Generally, lower thoracic esophageal cancer has a higher incidence of abdominal LNM than middle thoracic esophageal cancer [9]. However, could the rule still work in a wide extension of middle esophageal cancer? Does middle thoracic esophageal cancer with a lower margin invading the lower esophagus have the same risk of abdominal LNM as middle thoracic esophageal cancer without a lower margin invading the lower esophagus? How does it compare with lower thoracic esophageal cancer?

This study aimed to determine the value of the lower tumor margin in the prediction of abdominal LNM and stratify the risk of abdominal LNM based on the lower tumor margin (LTM).

## 2. Method

A retrospective review of patients who underwent esophagectomy for esophageal cancer from 2014 to 2017 in West China Hospital was performed. A barium esophagogram was used to locate the lower tumor margin. Therefore, only patients who underwent the examination before surgery in our hospital were enrolled. However, patients were excluded when the lower tumor margin was unable to be located on a barium esophagogram. Considering that only 69 patients received neoadjuvant chemotherapy, patients who received neoadjuvant therapy were also excluded. ESCC is the predominant histological type in China. In consideration of differences in biological characteristics among different histology types, only ESCC was included. The study was approved by the institutional review board of West China Hospital, Sichuan University. Informed consent was waived owing to the retrospective nature.

### 2.1. Lymph Node Grouping

According to the 8th edition AJCC staging criteria for cancer of the esophagus and EGJ, there are 5 abdominal lymph node stations and 15 thoracic lymph node stations. The abdominal lymph node stations included 16, 17, 18, 19, and 20 lymph node stations. The thoracic lymph stations included 2L, 2R, 8U, 4L, 4R, 5, 6, 7, 8M, 8Lo, 9L, 9R, 10L, 10R, and 15 stations.

### 2.2. Localization of the Lower Tumor Margin on the Barium Esophagogram

In this study, the distance between the lower tumor margin and esophagogastric junction (LED) was used to define the location of the lower tumor margin.

The growth of esophageal tumors destroys the esophageal wall and results in abnormal signs on esophagography. The most common signs include luminal narrowing, filling defect, mucosal irregularity, intraluminal mass, and ulceration. The start and end points of abnormal signs represent the upper and lower margins of the tumor, respectively.

**Figure 1:**
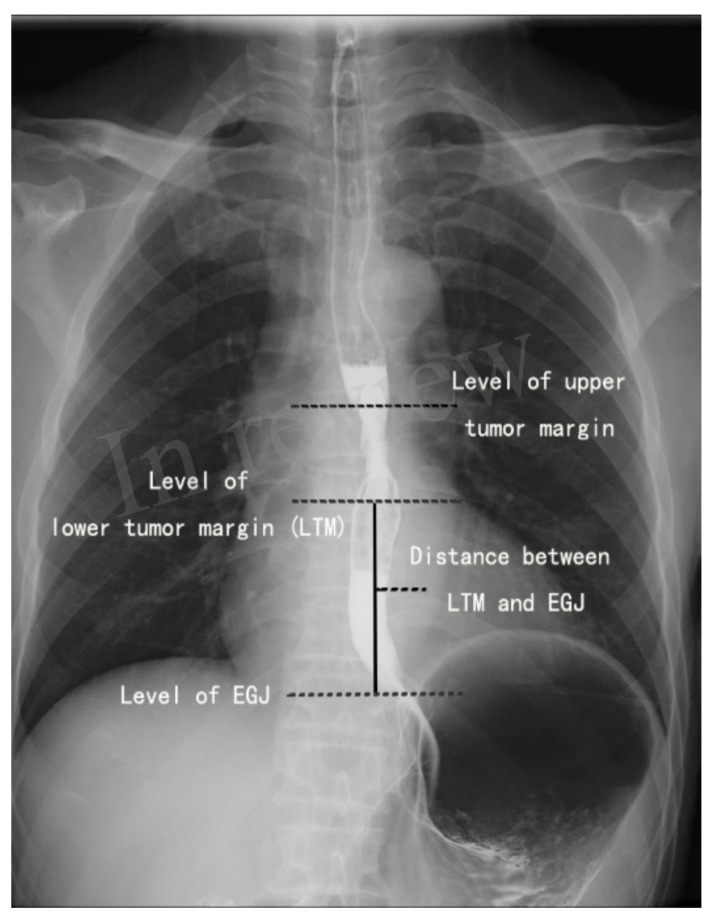
Measurement of the distance between the lower tumor margin and esophagogastric junction.

For patients with esophageal insufficiency or obstruction, esophageal endoscopy may not be able to obtain information about distal tumor involvement. We chose UGCXR to obtain complete information about the tumor location. First, based on the abnormal signs on esophagography, the tumor was located. Then, the image of esophagogram was enlarged so that we could mark the endpoint of the abnormal signs precisely and conveniently. After that, the distance between the LTM and EGJ could be measured easily. The measurements were performed on an anteroposterior barium esophagogram, as shown in Figure 1. The work was accomplished by a single author independently.

### 2.3. Statistical Analysis

All statistical analyses were run by SPSS 22.0 (IBM, Armonk, NY, USA). The chi-square test or Fisher’s exact test was used to detect whether a statistically significant difference appeared in categorical parameters. Kaplan–Meier with log-rank test was used to compare overall survival curves. For continuous data, Student’s *t* test or the Mann–Whitney U test was performed. The parameters with a *p* value of less than 0.1 in univariate analyses were included in multivariate logistic regression analysis to identify the risk factors for LNM. A PSM analysis was performed among patients with different LTM when multivariate analysis indicated that it was a risk factor for abdominal LNM. Therefore, we could remove bias from confounding factors and compare the risk of abdominal LNM according to the lower tumor margin directly. The selected groups were matched at a ratio of 1:1 using the nearest-neighbor method, and the match tolerance was set at 0.01 or less. Receiver operating characteristic curves and nomograms were applied to complete the prediction model. *p* values of less than 0.05 were considered statistically significant.

## 3. Result

A total of 846 patients with ESCC were enrolled. The total abdominal LNM rate was 48.29% (410 patients), while 70, 510, and 266 patients suffered LNM in the upper, middle, and lower thoracic esophagus, respectively.

### 3.1. Risk Factor for Abdominal LNM in Middle Thoracic ESCC

A total of 510 patients were diagnosed with middle ESCC. Among them, 143 patients had abdominal LNM, 177 had thoracic LNM, and 82 patients had LNM in both regions. Most characteristics were comparable between patients with abdominal LNM and patients without LNM (Table 1). However, the abdominal LNM group had significantly poorer differentiation and late T stage (*p* < 0.001). For LED, a statistical significance was found between the abdominal LNM group and the no-LNM group (*p* = 0.012).

According to the 8th edition AJCC staging criteria for cancer of the esophagus and EGJ, the length of the lower thoracic esophagus is usually 10 cm. To determine whether there is an effect on abdominal lymph node metastasis when the lower edge of middle esophageal cancer invades the lower esophagus, we came up with a cutoff of 10 cm for LED.

Then, multivariate logistic regression analysis was performed, as shown in Table 2. Differentiation grade, T stage and LED were identified as risk factors for abdominal LNM (*p* < 0.01). Patients with an LED less than 10 cm had a significantly higher risk of abdominal LNM (*p* = 0.006), as did poor differentiation or late T stage.

LTM was recognized as a risk factor for middle thoracic ESCC, so we compared patients with an LED less than 10 cm and those with an LED greater than 10 cm (Table 3). In total, 385 patients had an LED less than 10 cm, and the abdominal LNM rate was 32.2%. In contrast, 125 patients had an LED greater than 10 cm, and the rate was 15.2%, and the difference in abdominal LNM rate was found to be significant (*p* < 0.001). However, several variables were unbalanced between them. Then, we performed PSM and found that the difference remained significant, with rates of 30.2% and 15.5% (*p* = 0.008) (Supplementary Table S1). The variables included in the PSM included differentiation, T stage, N stage, gender, and proportion of drinking patients.

### 3.2. Risk Factor for Abdominal LNM in Lower Thoracic ESCC

A total of 266 patients had lower thoracic ESCC: 113 patients had abdominal LNM, 86 had thoracic LNM, and 58 had both. A comparison between patients with abdominal LNM and without any LNM is shown in Table 4. The abdominal LNM group had significantly poorly differentiated tumors, late T stage, and shorter LED than the no-LNM group (2.17 cm versus 2.79 cm, *p* = 0.041), which were all statistically significant. Moreover, the abdominal LNM group was more inclined to have EGJ invasion (41.6% versus 22.4%, *p* = 0.001). Other parameters were comparable between the two groups.

Six parameters were found to have p values less than 0.1 in the baseline comparison. Owing to some overlap of tumors invading the EGJ and LED, especially for lower ESCC, we only left the variable of tumors invading the EGJ. Multivariate logistic regression analysis was performed with the five parameters and found that late T stage and invasion of the EGJ were risk factors for abdominal LNM in lower thoracic ESCC (Table 5).

### 3.3. Comparison of Abdominal LNM between Middle Thoracic ESCC with LED Less Than 10 cm and Lower Thoracic ESCC without EGJ Invasion

There were 385 middle thoracic ESCC patients with an LED less than 10 cm, and the rate of abdominal LNM was 32.2%. For lower thoracic ESCC, 188 patients had a tumor that did not invade the EGJ, and the rate of abdominal LNM was 35.1%. The rate of abdominal LNM was comparable between the two groups (*p* = 0.489, Table 6). However, the distributions of several factors that might have strong relationships with LNM were not comparable. Then, PSM analysis was performed. The variables included in the PSM included gender, age, proportion of drinking patients, smoking patients, degree of differentiation, T stage, and N stage. One hundred sixty-seven pairs of patients were successfully matched. The confounding factors were well balanced in the PSM cohort (Supplementary Table S2). The rate of abdominal LNM remained comparable between middle thoracic ESCC with an LED less than 10 cm and lower thoracic ESCC without invading the EGJ (*p* = 0.424).

### 3.4. Abdomen LNM Risk Stratification for ESCC according to LTM

As shown in Table 7, upper ESCC had an abdominal LNM rate of 2.9%, which belonged to the very-low-risk group, while middle ESCC with LED more than 10 cm was 15.2% and was classified as the low-risk group. Middle ESCC with LED less than 10 cm had an abdominal LNM rate of 32.2%, which was similar to lower ESCC without EGJ invasion (35.1%), and both were classified as the moderate-risk group, while lower ESCC with EGJ invasion was classified as the high-risk group by a rate of 60.3%.

### 3.5. Abdominal LNM Prediction Model for ESCC

Tumor location, differentiation grade, pathological T stage, and LED were risk factors for LNM from the above results; enlarged lymph nodes in preoperative CT scans were also related to LNM. Therefore, we set a prediction model of abdominal LNM with the above five variables. Patients were randomly divided into a modeling group (D set) and a verifying group (V set) at a 1:1 ratio. The ROC curve of the prediction model is shown in Supplementary Figure S1. The AUCs of model 1 (full model) and model 2 (stepwise model) fluctuated between 0.75–0.77. Based on the prediction model, we obtained the nomogram in Supplementary Figure S2. The prediction model demonstrated that the probability of abdominal LNM exceeded 90% when the total score exceeded 250 points. However, the probability was less than 10% when the total score was less than 190 points.

### 3.6. Long-Term Survival and Abdominal LNM in ESCC

In total, 619 eligible patients were included in the survival analysis. There were 123, 73, and 111 patients characterized with purely thoracic LNM, purely abdominal LNM, and both-region LNM, respectively. The OS of patients without LNM was significantly better than that of others. The OS of patients with LNM in only one region was better than that of patients with LNM in both regions (*p* < 0.05), while the OS of patients with purely abdominal LNM was comparable to that of patients with purely thoracic LNM (*p* = 0.12) (Supplementary Figure S3). Concerning N1 patients, differences existed between the three groups (*p* = 0.023), and the OS of either the purely abdominal LNM group or the thoracic LNM group was significantly better than that of the group with both. However, the groups with single-region LNM were still comparable to each other (*p* = 0.014), as shown in Supplementary Figure S4. For N2-N3 patients, all three groups were similar, as shown in Supplementary Figure S5.

## 4. Discussion

The incidence of abdominal lymph node recurrence in patients with esophageal cancer is 8.4–20.0%. In addition, our study also found that patients with more extensive lymph node metastasis had lower long-term survival. Therefore, thorough abdominal lymph node dissection is closely related to prognosis, and more lymph nodes can also be dissected to obtain more accurate N staging after surgery. It is essential to evaluate the risk of abdominal LNM preoperatively to guide the intraoperative dissection strategy. Due to the characteristics of longitudinal and bidirectional drainage of esophageal lymph nodes, tumor location is closely related to LNM risk in patients with esophageal cancer [8,10,11,12]. Chen et al. demonstrated that the abdominal LNM incidence of upper, middle, and lower ESCC increased successively, reaching 8.0%, 27.2%, and 51.7%, respectively [13]. Hagens et al. found that the abdominal LNM incidence in upper, middle, and lower ESCC was 6%, 20%, and 29%, respectively [14]. In this study, a total of 846 patients with ESCC were included. The overall incidence of abdominal LNM was 30.5%, and the abdominal LNM rates of upper, middle, and lower ESCC were 2.9%, 28.0%, and 42.5%, respectively. Indeed, tumor location is an important indicator of abdominal LNM risk. Other studies also find that LNM risk and prognosis were different for different location of esophageal cancer [15,16,17]. Our study further found that patients with esophageal cancer at the same location were defined by the tumor center. When the lower edge of the tumor was located at a different site, the risk of abdominal LNM was still different. According to the UGCXR of middle and lower ESCC, LED showed significant differences between the abdominal LNM group and the non-LNM group. Logistic regression analysis indicated that LTM involving the EGJ was a risk factor for abdominal LNM in patients with lower ESCC, while LED less than 10 cm was a risk factor for abdominal LNM in patients with middle ESCC, and there was no significant difference in the risk of abdominal LNM between middle ESCC patients with LED less than 10 cm and lower ESCC patients without EGJ involvement. However, node skip metastasis was also frequently found in esophageal cancer patients [18,19,20], and we have to take node skip metastasis into account except consideration of tumor location and check preoperative CT scan for LNM risk carefully.

Abdominal LNM incidence in lower ESCC patients with EGJ involvement was as high as 60.3%, which was the highest risk group, and the incidence was 35.1% in patients with lower ESCC without involving the EGJ. Regarding middle esophageal cancer patients with LED less than 10 cm, the incidence of abdominal LNM was 32.2%, which was close to lower ESCC without EGJ involvement, and both belonged to the moderate risk group. In middle esophageal cancer patients with LED greater than 10 cm, the incidence of abdominal LNM decreased to 15.2%, which belonged to the low-risk group. The abdominal LNM rate of patients with upper esophageal cancer was only 2.9%, which was categorized to the very-low-risk group. According to the location of the tumor lower edge, patients with a high or low risk of abdominal LNM can be better distinguished for the center-based same location of esophageal cancer. Similarly, one study also divided patients into 3 groups based on the distance of tumor’s proximal edge to esophagogastric junction (low; ≤2 cm, medium; 2.0–7.0 cm, and high; >7.0 cm) and found that paratracheal LN metastases were more frequent with the proximal tumors [21].

Mine et al. enrolled patients with lower esophageal cancer and gastroesophageal junction cancer and found that the incidence of LNM in the upper thoracic area was only 3.3% when the upper edge of the tumor was below the foramen vena cava, and when the upper edge of the tumor was beyond the foramen vena cava, the incidence of LNM in the middle and upper thoracic areas was 37.8% and 36.4%, respectively [22]. Therefore, the position of the upper tumor edge could be used as an important indicator of middle and upper thoracic LNM risk for lower esophageal cancer. Ueda et al. measured the distance between the lower tumor edge, tumor center, upper tumor edge, and gastroesophageal junction in surgically removed specimens and found that the distance between the lower tumor edge and gastroesophageal junction is more valuable in predicting abdominal lymph node metastasis than the distance from the tumor center to the gastroesophageal junction [9]. Other studies also found the LNM risk is related to distance for EGJ cancer [23,24,25]. The results of our study could better help stratify abdominal LNM risk before surgery via LED.

Some limitations exist in this study. First, it is difficult to locate the lower tumor edge of early esophageal lesions in UGCXR. Second, the prediction model was not applicable for patients who received neoadjuvant therapy and esophageal adenocarcinoma, which needs to be further verified. Third, abdominal lymph nodes of esophageal cancer included groups 16, 17, 18, 19, and 20, but more than 95% of the abdominal LNM was concentrated in groups 16 and 17 in this study. Fourth, we determined the location of the tumor from a macroscopic point of view by imaging. However, the invasion of malignant cells can only be observed under the microscope to define the true edge of the tumor, which may differ from the location of the tumor on image.

## 5. Conclusions

LED measured by UGCXR was a risk factor for abdominal LNM. This distance was negatively correlated with the risk of abdominal LNM; that is, a smaller LED was accompanied by a higher risk of abdominal LNM. According to the location of LTM, the abdominal LNM risk of ESCC was stratified as follows: the lower margin of lower ESCC involving the EGJ (high-risk group) > the lower margin of lower ESCC not involving the EGJ (moderate-risk group) = the middle ESCC with LED less than 10 cm (moderate-risk group) > the middle ESCC with LED greater than 10 cm (low-risk group) > the upper ESCC (very-low-risk group). The abdominal LNM prediction model based on the findings could well assess abdominal LNM risk in ESCC patients preoperatively and could guide the strategy of intraoperative abdominal lymph node dissection well. In ESCC patients, abdominal LNM was as important as thoracic LNM concerning OS.

## Figures and Tables

**Table 1:** Characteristics of middle ESCC patients with abdominal LNM and patients without any LNM.

Variable	Middle ESCC with Abdomen LNM	Middle ESCC without LNM	*p* Value
Sex			0.186
Male	115 (80.4%)	203 (74.6%)	
Female	28 (19.6%)	69 (25.4%)	
Age (years)	61.99 ± 8.05	61.88 ± 8.01	0.897
BMI (kg/m^2^)	21.80 ± 2.63	22.33 ± 3.07	0.079
Preoperative dysphagia duration (month)	3.35 ± 2.88	3.22 ± 3.69	0.716
Smoking			0.193
Yes	94 (65.7%)	161 (59.2%)	
No	49 (34.3%)	111 (40.8%)	
Drinking			0.228
Yes	83 (58.0%)	141 (51.8%)	
No	60 (42.0%)	131 (48.2%)	
Diabetes			0.404
Yes	7 (4.9%)	19 (7.0%)	
No	136 (95.1%)	253 (93.0%)	
Hypertension			0.197
Yes	22 (15.4%)	56 (20.6%)	
No	121 (84.6%)	216 (79.4%)	
Differentiation grade			<0.001
High	2 (1.4%)	15 (5.5%)	
Moderate	58 (40.6%)	155 (57.0%)	
Low	83 (58.0%)	102 (37.5%)	
p-T stage			<0.001
Tis	0 (0.0%)	6 (2.2%)	
T1	8 (5.6%)	53 (19.5%)	
T2	24 (16.8%)	64 (23.5%)	
T3	92 (64.3%)	129 (47.4%)	
T4a	19 (13.3%)	20 (7.4%)	
LED (cm)	7.55 ± 2.33	8.21 ± 2.65	0.012

**Table 2:** Multivariate logistic regression analysis of abdominal LNM in middle ESCC.

Variable	Wald c^2^ Value	OR	95% CI	*p* Value
LED < 10 cm				0.006
No	ref			
Yes	7.574	2.250	1.263–4.007	
BMI ≥ 24 kg/m^2^				0.276
No	ref			
Yes	1.184	0.758	0.460–1.248	
Differentiation grade				<0.001
Moderate-high	Ref			
Low	15.466	2.372	1.542–3.647	
p-T stage				<0.001
Tis-T2	Ref			
T3-T4a	18.122	2.807	1.745–4.515	

**Table 3:** Characteristics between groups with short LEDs and long LEDs.

Variable	LED < 10 cm	LED > 10 cm	*p* Value
Sex			0.027
Male	290 (75.3%)	106 (84.8%)	
Female	95 (24.7%)	19 (15.2%)	
Age (years)	61.79 ± 8.08	61.99 ± 8.29	0.812
BMI (kg/m^2^)	22.22 ± 2.98	22.31 ± 3.28	0.795
Smoking			0.388
Yes	236 (61.3%)	82 (65.6%)	
No	149 (38.7%)	43 (34.4%)	
Drinking			0.003
Yes	201 (52.2%)	84 (67.2%)	
No	184 (47.8%)	41 (32.8%)	
Diabetes			0.777
Yes	22 (5.7%)	8 (6.4%)	
No	363 (94.3%)	117 (93.6%)	
Hypertension			0.395
Yes	78 (20.3%)	21 (16.8%)	
No	307 (79.7%)	104 (83.2%)	
differentiation			0.869
High	12 (3.1%)	5 (4.0%)	
Moderate	191 (49.6%)	60 (48.0%)	
Low	182 (47.3%)	60 (48.0%)	
p-T stage			0.015
Tis	3 (0.8%)	3 (2.4%)	
T1	40 (10.4%)	25 (20.0%)	
T2	80 (20.8%)	26 (20.8%)	
T3	215 (55.8%)	63 (50.4%)	
T4a	47 (12.2%)	8 (6.4%)	
p-N stage			0.337
N0	198 (51.4%)	74 (59.2%)	
N1	99 (25.7%)	31 (24.8%)	
N2	66 (17.1%)	16 (12.8%)	
N3	22 (5.7%)	4 (3.2%)	
Abdomen LNM			<0.001
Yes	124 (32.2%)	19 (15.2%)	
No	261 (67.8%)	106 (84.8%)	

**Table 4:** Characteristics of patients with abdominal LNM and without LNM in lower ESCC.

Variable	Lower ESCC with Abdomen LNM	Lower ESCC without LNM	*p* Value
Sex			0.292
Male	93 (82.3%)	109 (87.2%)	
Female	20 (17.7%)	16 (12.8%)	
Age (year)	60.96 ± 9.67	62.87 ± 7.80	0.097
BMI (kg/m^2^)	22.08 ± 2.95	22.80 ± 2.99	0.062
Smoking			0.837
Yes	80 (70.8%)	90 (72.0%)	
No	33 (29.2%)	35 (28.0%)	
Drinking			0.214
Yes	80 (70.8%)	79 (63.2%)	
No	33 (29.2%)	46 (36.8%)	
Diabetes			0.418
Yes	6 (5.3%)	4 (3.2%)	
No	107 (94.7%)	121 (96.8%)	
Hypertension			0.878
Yes	29 (25.7%)	31 (24.8%)	
No	84 (74.3%)	94 (75.2%)	
Differentiation grade			0.009
High	0 (0.0%)	10 (8.0%)	
Moderate	59 (52.2%)	62 (49.6%)	
Low	54 (47.8%)	53 (42.4%)	
p-T stage			<0.001
Tis	0 (0.0%)	3 (2.4%)	
T1	4 (3.5%)	24 (19.2%)	
T2	21 (18.6%)	30 (24.0%)	
T3	83 (73.5%)	67 (53.6%)	
T4a	5 (4.4%)	1 (0.8%)	
LED (cm)	2.17 ± 2.32	2.79 ± 2.33	0.041
LED = 0 cm			0.001
Yes	47 (41.6%)	28 (22.4%)	
No	66 (58.4%)	97 (77.6%)	

**Table 5:** Multivariate logistic regression analysis of abdominal LNM in lower ESCC.

Variable	Wald c^2^ Value	OR	95% CI	*p* Value
LED = 0 cm				0.004
No	Ref			
Yes	8.143	2.326	1.303–4.155	
Age				0.339
≤65	ref			
>65	0.915	0.764	0.441–1.326	
BMI (kg/m^2^)				0.898
<24	ref			
≥24	0.016	0.961	0.525–1.761	
Differentiation grade				0.491
Low	Ref			
Moderate-high	0.473	0.828	0.483–1.419	
p-T stage				<0.001
Tis-T2	Ref			
T3-T4a	12.479	2.850	1.594–5.097	

**Table 6:** Abdominal LNM between middle thoracic ESCC with LED less than 10 cm and lower thoracic ESCC without EGJ invasion.

Variable	Middle ESCC with LED < 10 cm	Lower ESCC with LED > 10 cm	*p* Value
Sex			0.055
Male	290 (75.3%)	155 (82.4%)	
Female	95 (24.7%)	33 (17.6%)	
Age (year)	61.79 ± 8.08	61.98 ± 8.54	0.799
BMI (kg/m^2^)	22.39 ± 2.94	22.22 ± 2.98	0.527
Smoking			0.027
Yes	236 (61.3%)	133 (70.7%)	
No	149 (38.7%)	55 (29.3%)	
Drinking			0.003
Yes	201 (52.2%)	123 (65.4%)	
No	184 (47.8%)	65 (34.6%)	
Diabetes			0.307
Yes	22 (5.7%)	7 (3.7%)	
No	363 (94.3%)	181 (96.3%)	
Hypertension			0.388
Yes	78 (20.3%)	44 (23.4%)	
No	307 (79.7%)	144 (76.6%)	
Differentiation grade			0.780
High	12 (3.1%)	8 (4.3%)	
Moderate	191 (49.6%)	93 (49.5%)	
Low	182 (47.3%)	87 (46.3%)	
p-T stage			0.826
Tis	3 (0.8%)	3 (1.6%)	
T1	40 (10.4%)	26 (13.8%)	
T2	80 (20.8%)	38 (20.2%)	
T3	215 (55.8%)	114 (60.6%)	
T4a	47 (12.2%)	7 (3.7%)	
p-N stage			0.015
N0	198 (51.4%)	97 (51.6%)	
N1	99 (25.7%)	53 (28.2%)	
N2	66 (17.1%)	27 (14.4%)	
N3	22 (5.7%)	11 (5.9%)	
Abdomen LNM			0.489
Yes	124 (32.2%)	66 (35.1%)	
No	261 (67.8%)	122 (64.9%)	

**Table 7:** Abdomen LNM risk stratification.

Location of Lower Tumor Margin	Abdomen LNM Rate	Abdomen LNM Risk
Upper esophagus	2.9%	Very low
Middle esophagus with LED more than 10 cm	15.2%	Low
Middle esophagus with LED less than 10 cm	32.2%	
Lower esophagus without invading EGJ	35.1%	Moderate
Lower esophagus with invading EGJ	60.3%	High

## Data Availability

Data from this study can be acquired from the corresponding author upon appropriate request.

## Supplementary Materials

The following supporting information can be downloaded at: https://www.mdpi.com/article/10.3390/jcm12072657/s1.

## Abbreviations

ESCC—esophageal squamous cell carcinoma, UGCXR—upper gastrointestinal contrast-enhanced X-ray, LNM—lymph node metastasis, EGJ—esophagogastric junction, LTM—lower tumor margin, LED—distance between lower tumor margin and esophagogastric junction, OS—overall survival, PSM—propensity score match, BMI—body mass index, p-T—pathological tumor, cm: centimeter, AUC—area under curve, ROC—receiver operating characteristics curve, OR—odds ratio.
