# Supplementary material for: Role of Lower Esophageal Squamous Cell Carcinoma Margin Location on Abdominal Lymph Node Metastasis Risk
Source: J Clin Med. 2023 Apr 3;12(7):2657. doi: 10.3390/jcm12072657 (PMC10095315; doi:10.3390/jcm12072657)
Supplement: Supplementary file 1 [file jcm-12-02657-s001.zip › jcm-2201520-supplementary.pdf]

Supplementary Table S1. Characteristics between groups with short LEDs and long LEDs.

Variable	Before PSM			After PSM		
	LED<10 cm	LED>10 cm	p value	LED<10 cm	LED>10 cm	p value
Sex			0.027			0.851
Male	290 (75.3%)	106 (84.8%)		99 (85.3%)	100 (86.2%)	
Female	95 (24.7%)	19 (15.2%)		17 (14.7%)	16 (13.8%)	
Age (years)	61.79±8.08	61.99±8.29	0.812	61.14±8.35	61.78±8.36	0.562
BMI (kg/m2)	22.22±2.98	22.31±3.28	0.795	22.18±2.57	22.15±3.01	0.941
Smoking			0.388			1.000
Yes	236 (61.3%)	82 (65.6%)		79 (68.1%)	79 (68.1%)	
No	149 (38.7%)	43 (34.4%)		37 (31.9%)	37 (31.9%)	
Drinking			0.003			0.671
Yes	201 (52.2%)	84 (67.2%)		78 (67.2%)	81 (69.8%)	
No	184 (47.8%)	41 (32.8%)		38 (32.8%)	35 (30.2%)	
Diabetes			0.777			0.789
Yes	22 (5.7%)	8 (6.4%)		7 (6.0%)	8 (6.9%)	
No	363 (94.3%)	117 (93.6%)		109(94.0%)	108(93.1%)	
Hypertension			0.395			0.253
Yes	78 (20.3%)	21 (16.8%)		27 (23.3%)	20 (17.2%)	
No	307 (79.7%)	104 (83.2%)		89 (76.7%)	96 (82.8%)	
differentiation			0.869			0.847
High	12 (3.1%)	5 (4.0%)		4 (3.4%)	5 (4.3%)	

Moderate	191 (49.6%)	60 (48.0%)		51 (44.0%)	54 (46.6%)	
Low	182 (47.3%)	60 (48.0%)		61 (52.6%)	57 (49.1%)	
p-T stage			0.015			0.951
Tis	3 (0.8%)	3 (2.4%)		2 (1.7%)	3 (2.6%)	
T1	40 (10.4%)	25 (20.0%)		23 (19.8%)	21 (18.1%)	
T2	80 (20.8%)	26 (20.8%)		24 (20.7%)	24 (20.7%)	
T3	215 (55.8%)	63 (50.4%)		63 (54.3%)	62 (53.4%)	
T4a	47 (12.2%)	8 (6.4%)		4 (3.4%)	6 (5.2%)	
p-N stage			0.337			0.961
N0	198 (51.4%)	74 (59.2%)		71 (61.2%)	71 (61.2%)	
N1	99 (25.7%)	31 (24.8%)		29 (25.0%)	28 (24.1%)	
N2	66 (17.1%)	16 (12.8%)		11 (9.5%)	13 (11.2%)	
N3	22 (5.7%)	4 (3.2%)		5 (4.3%)	4 (3.4%)	
Abdomen LNM			<0.001			0.008
Yes	124 (32.2%)	19 (15.2%)		35 (30.2%)	18 (15.5%)	
No	261 (67.8%)	106 (84.8%)		81 (69.8%)	98 (84.5%)	

Supplementary Table S2. Abdominal LNM between middle thoracic ESCC with LED less than 10 cm and lower thoracic ESCC without EGJ invasion.

Variable	Before PSM			After PSM		
	Middle ESCC with LED<10 cm	Lower ESCC With LED>0 cm	P value	Middle ESCC With LED<10 cm	Lower ESCC With LED>0 cm	p value
Sex			0.055			0.663
Male	290 (75.3%)	155 (82.4%)		140 (83.8%)	137 (82.0%)	
Female	95 (24.7%)	33 (17.6%)		27 (16.2%)	30 (18.0%)	
Age (year)	61.79±8.08	61.98±8.54	0.799	61.91±7.93	61.83±8.67	0.932
BMI (kg/m2)	22.39±2.94	22.22±2.98	0.527	22.57±2.89	22.34±2.92	0.474
Smoking			0.027			0.903
Yes	236 (61.3%)	133 (70.7%)		119 (71.3%)	120 (71.9%)	
No	149 (38.7%)	55 (29.3%)		48 (28.7%)	47 (28.1%)	
Drinking			0.003			0.817
Yes	201 (52.2%)	123 (65.4%)		111 (66.5%)	109 (65.3%)	
No	184 (47.8%)	65 (34.6%)		56 (33.5%)	58 (34.7%)	
Diabetes			0.307			0.428
Yes	22 (5.7%)	7 (3.7%)		9 (5.4%)	6 (3.6%)	
No	363 (94.3%)	181 (96.3%)		158 (94.6%)	161 (96.4%)	
Hypertension			0.388			0.608
Yes	78 (20.3%)	44 (23.4%)		42 (25.1%)	38 (22.8%)	

No	307 (79.7%)	144 (76.6%)		125 (74.9%)	129 (77.2%)	
differentiation			0.780			0.436
High	12 (3.1%)	8 (4.3%)		3 (1.8%)	7 (4.2%)	
Moderate	191 (49.6%)	93 (49.5%)		88 (52.7%)	85 (50.9%)	
Low	182 (47.3%)	87 (46.3%)		76 (45.5%)	75 (44.9%)	
p-T stage			0.015			0.934
Tis	3 (0.8%)	3 (1.6%)		1 (0.6%)	2 (1.2%)	
T1	40 (10.4%)	26 (13.8%)		21 (12.6%)	23 (13.8%)	
T2	80 (20.8%)	38 (20.2%)		33 (19.8%)	30 (18.0%)	
T3	215 (55.8%)	114 (60.6%)		103 (61.7%)	105 (62.9%)	
T4a	47 (12.2%)	7 (3.7%)		9 (5.4%)	7 (4.2%)	
p-N stage			0.826			0.695
N0	198 (51.4%)	97 (51.6%)		82 (49.1%)	80 (47.9%)	
N1	99 (25.7%)	53 (28.2%)		43 (25.7%)	51 (30.5%)	
N2	66 (17.1%)	27 (14.4%)		28 (16.8%)	26 (15.6%)	
N3	22 (5.7%)	11 (5.9%)		14 (8.4%)	10 (6.0%)	
Abdomen LNM			0.489			0.424
Yes	124 (32.2%)	66 (35.1%)		56 (33.5%)	63 (37.7%)	
No	261 (67.8%)	122 (64.9%)		111 (66.5%)	104 (62.3%)	

Supplementary Figure S1

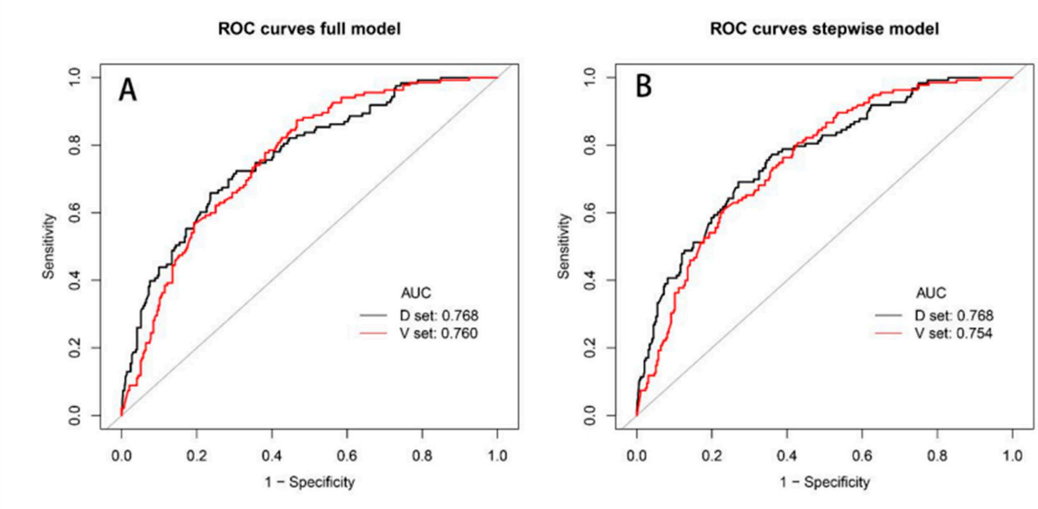

Esophageal squamous cell carcinoma abdominal lymph node metastasis prediction model Receiver operating characteristic curve (ROC curve)

Figure A: ROC curves of modeling group and validation group in model 1; Figure B: ROC curves of modeling group and validation group in model 2. AUC: Area under the curve; D set: modeling group; V set: Validation group

Supplementary Figure S2

Nomogram for evaluating the probability of abdominal lymph node metastasis in esophageal squamous cell carcinoma

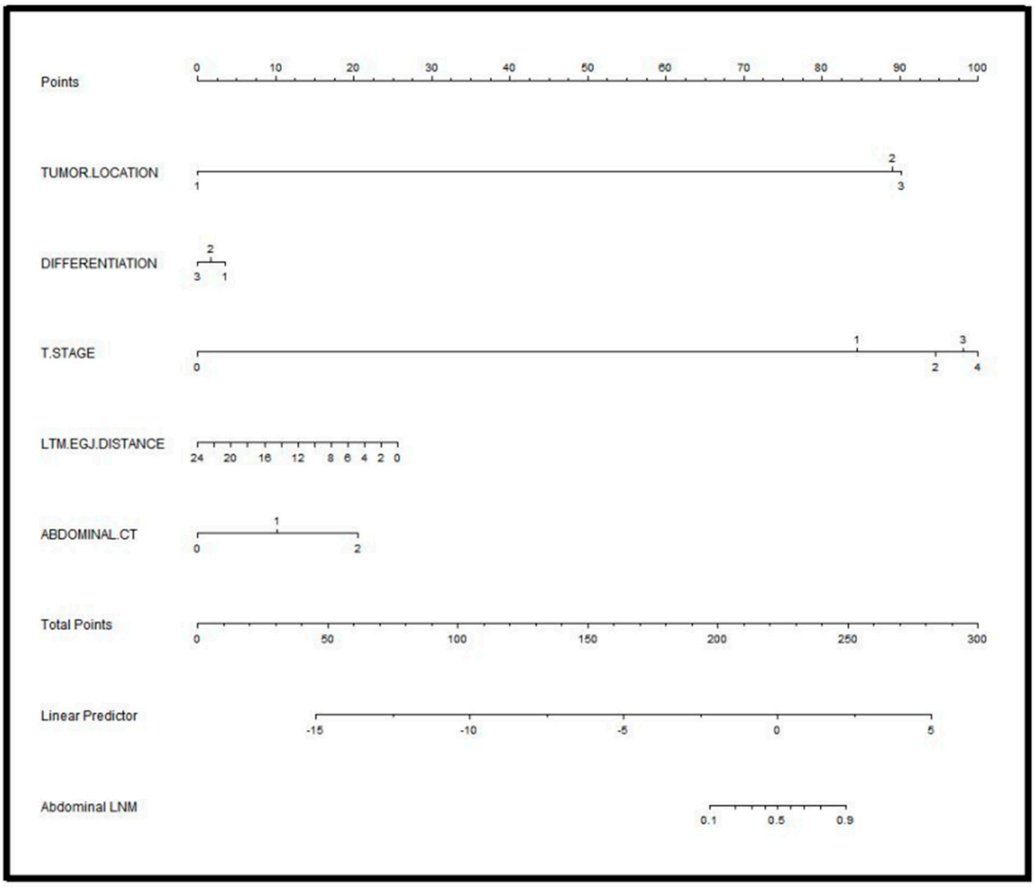

Tumor location: 1= upper, 2= middle, 3= lower; T Stage: 0=Tis stage, 1=T1 stage, 2=T2 stage, 3=T3 stage, 4=T4a stage; LTM.EGJ.Distance: the distance between the lower edge of the tumor and the gastroesophageal junction (cm); Abdominal CT: 0= no abdominal lymph node enlargement detected by abdominal CT, 1= abdominal lymph node enlargement with a diameter less than 1 cm, 2= abdominal lymph node enlargement with a diameter greater than 1 cm; Abdominal LNM: probability of abdominal lymph node metastasis.

Supplementary Figure S3

The overall survival (OS) curves of patients with different lymph node metastasis status

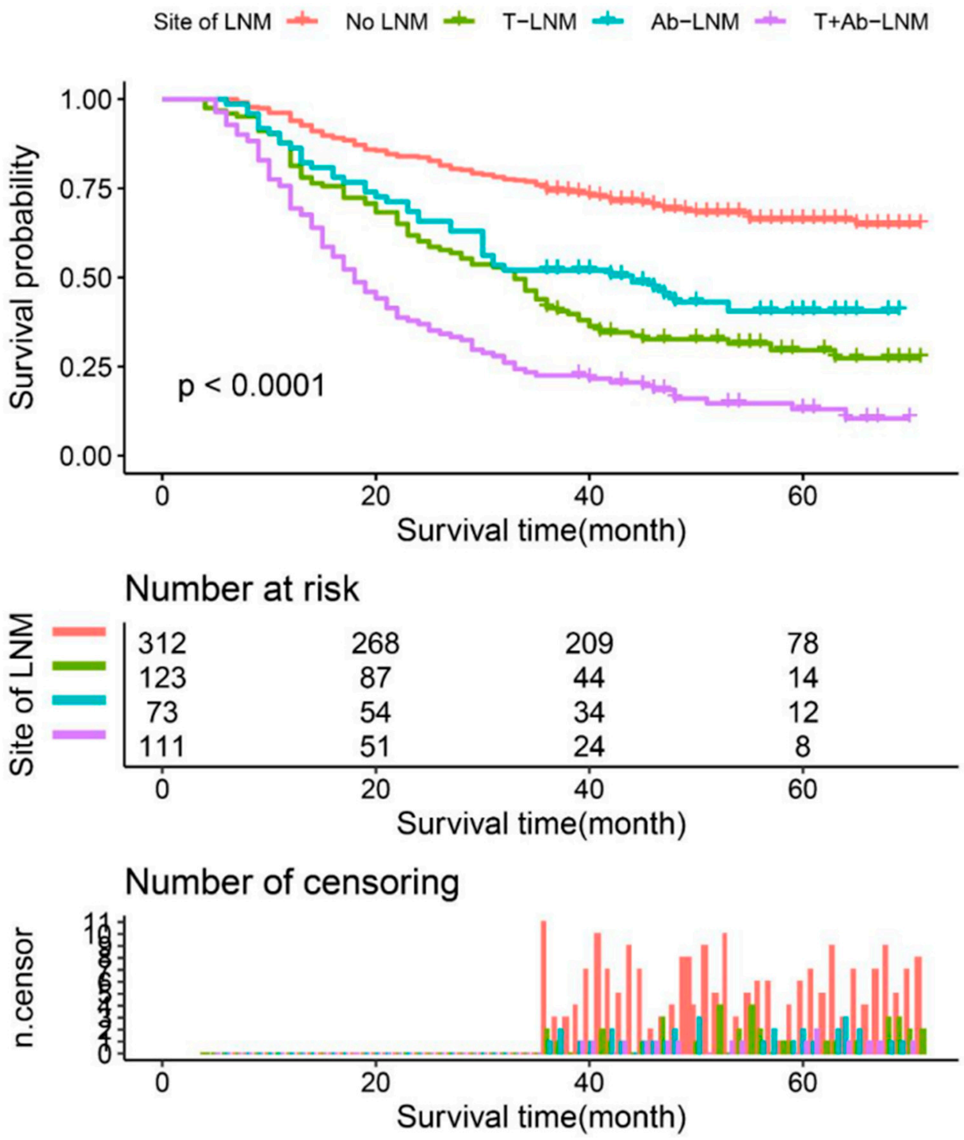

No LNM: no lymph node metastasis; T-LNM: only thoracic lymph node metastasis; Ab-LNM: only abdominal lymph nodes metastasis; Ab+T-LNM: thoracic and abdominal lymph node metastasis.

Supplementary Figure S4

The overall survival (OS) curves of N1 stage patients with different lymph node metastasis status

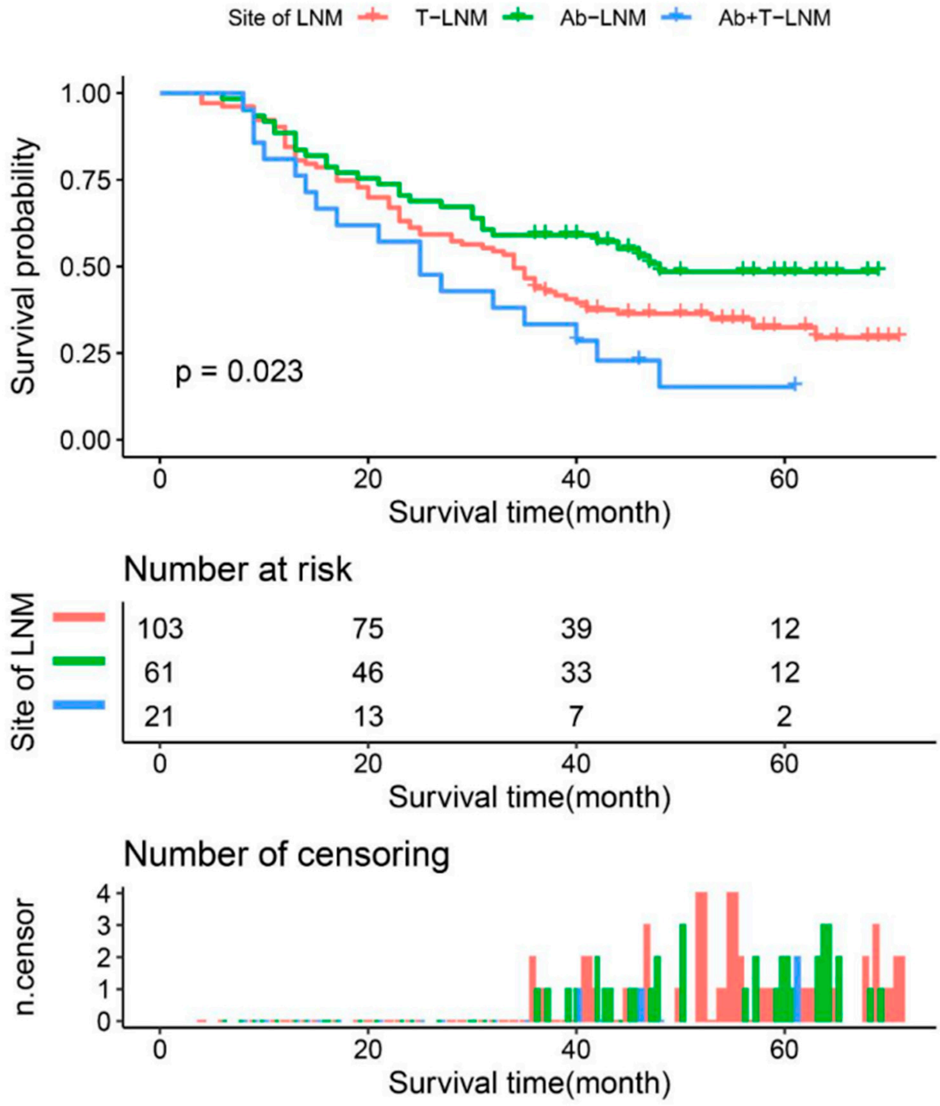

T-LNM: only thoracic lymph node metastasis group; Ab-LNM: only abdominal lymph node metastasis group; Ab+T-LNM: thoracic and abdominal lymph node metastasis group.

Supplementary Figure S5

The overall survival (OS) of stage N2-N3 patients with different lymph node metastasis status

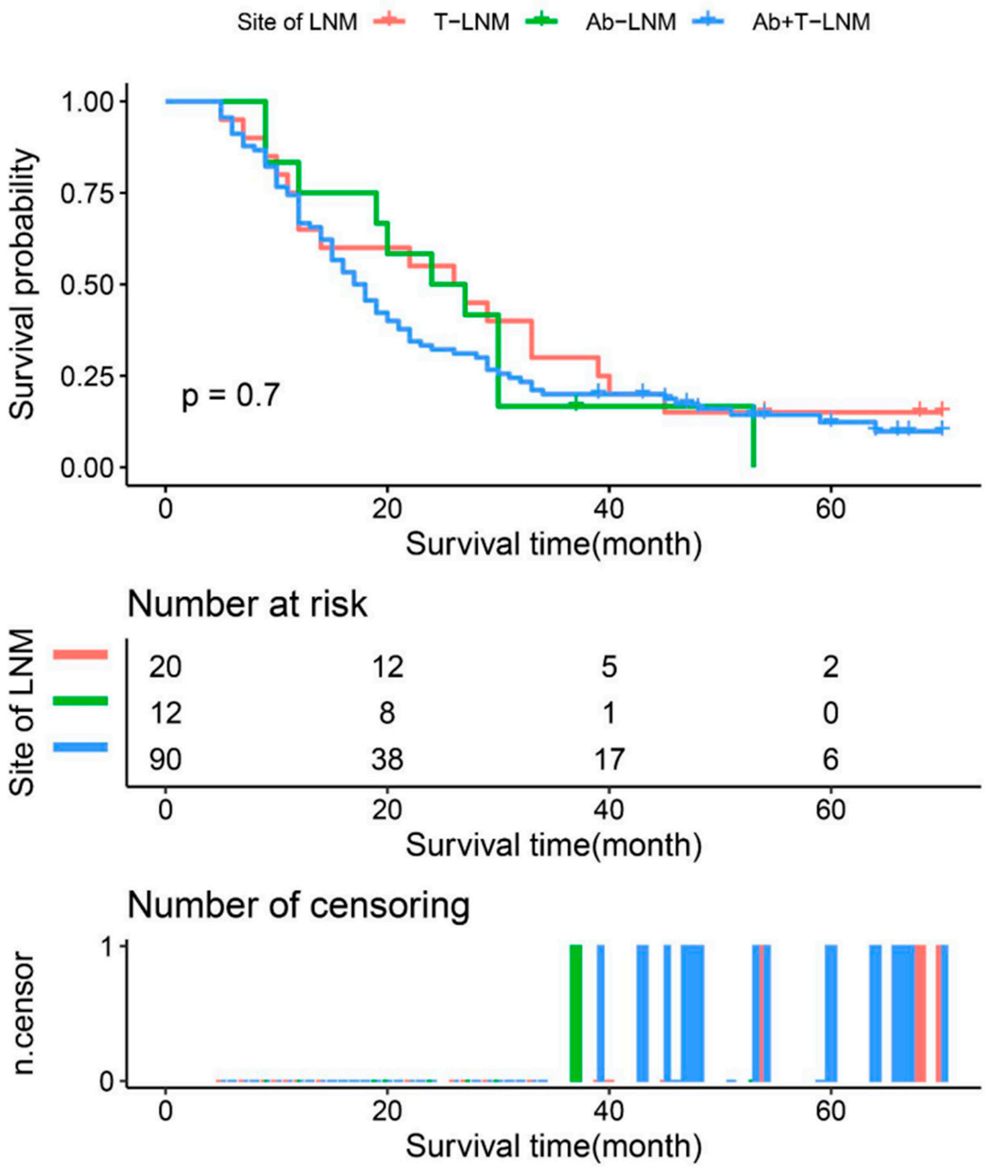

T-LNM: only thoracic lymph node metastasis group; Ab-LNM: only abdominal lymph node metastasis group; Ab+T-LNM: thoracic and abdominal lymph node metastasis group.
